# Child’s Developmental Disabilities and Parental Health in Later Life: Do Parental Race and Gender Matter?

**DOI:** 10.1093/geroni/igab046.1211

**Published:** 2021-12-17

**Authors:** Juha Lee, Manjing Gao, Chioun Lee

**Affiliations:** 1 University of California, Riverside, Anaheim, California, United States; 2 University of California, Riverside, Riverside, California, United States

## Abstract

Parents, particularly mothers, who experienced early life adversities (ELAs) are more likely to have a child with developmental disabilities (DD). We have little knowledge about how parental health varies across race-gender groups among those with a DD child and the role of ELAs in the associations. Using Black and White adults (n = 8,778; 25% Blacks) from the Midlife in the United States (MIDUS) study, we examine racial disparities in the impact of having a child with DD (vs. having healthy children) on parental health outcomes. This study questions (1) the extent to which parents’ ELAs (e.g., poverty and abuse) are associated with having a child with DD and (2) how considering early-life factors reveals racial and gender disparities in the impact of having a child with DD. We found that as the number of ELAs increases, the probability of having a healthy child decreases for all race-gender groups, but most dramatically for Black women. Having a DD has adverse effects on chronic illnesses and functional limitations more for mothers than fathers. Black women are most adversely affected, with no effect on Black men. There is no gender difference in the impact of having a DD child on depressive symptoms, yet White parents are more vulnerable than Black parents. After controlling for ELAs, the adverse effects of having a DD child on both physical and mental health remain significant. Future research should identify life-course circumstances that reveal why the impact of having a DD child varies by race and gender.

